# Lumbar Erector Spinae Plane Block for Total Hip Arthroplasty Comparing 24-Hour Opioid Requirements: A Randomized Controlled Study

**DOI:** 10.1155/2022/9826638

**Published:** 2022-10-03

**Authors:** Dahlia Townsend, Nasir Siddique, Atsumi Kimura, Yaacov Chein, Eli Kamara, John Pope, Mitchell Weiser, Singh Nair, Iyabo Muse

**Affiliations:** ^1^Montefiore Medical Center, Department of Anesthesiology, 111 E. 210th Street, Bronx, NY 10467, USA; ^2^University of Pittsburgh Medical Center Shadyside, Department of Anesthesiology and Perioperative Medicine, 5230 Centre Ave, Pittsburgh, PA 15232, USA; ^3^Nova Southeastern University, Dr. Kiran C. Patel College of Allopathic Medicine, 3200 S University Drive, Fort Lauderdale, FL 33328, USA; ^4^Montefiore Medical Center, Department of Orthopedic Surgery, Division of Joint Replacement, 111 E. 210th Street, Bronx, NY 10467, USA; ^5^Westchester Medical Center-New York Medical College, Department of Anesthesiology, 100 Woods Road, Valhalla, NY 10595, USA

## Abstract

**Design:**

Prospective, randomized, controlled trial. *Patients*. Sixty-three adult patients with an American Society of Anesthesiologists Status I–III who are undergoing elective primary total hip arthroplasty. *Interventions.* Patients were randomized to the control group (no block) or the ESPB group (preoperative ultrasound-guided lumbar ESPB). Intraoperatively, all patients received spinal anesthesia with moderate sedation. Postoperatively, patients received a standardized multimodal analgesia protocol. *Measurements*. The primary outcome was cumulative opioid consumption at 24 hours postoperatively. Secondary outcomes included cumulative opioid consumption at 8 hours and through 48 hours postoperatively and pain scores at 24 and 48 hours post surgery. *Main Results.* Thirty-one patients were randomized to the control group (spinal alone) and 32 patients to the ESPB group. The median opioid requirement in the first 8 hours after surgery was higher in the control group (28 mg of oral morphine equivalents (OME) versus 5 mg of OME in the ESPB group) (*p* = 0.013). There was no statistically significant difference in opioid consumption between the groups at 24 hours (*p* = 0.153) or 48 hours (*p* = 0.357) postoperatively. There was no statistically significant difference in pain scores between the two groups through 24 hours (*p* = 0.143) or 48 hours (*p* = 0.617) after surgery.

**Conclusion:**

Lumbar ESPB reduces opioid utilization during the first 8 hours postoperatively after total hip arthroplasty but not thereafter. Evaluating the use of either adding a local anesthetic adjunct to the ESPB or using longer-acting local anesthetic warrants further investigation.

## 1. Introduction

Approximately 500,000 hip arthroplasties are performed each year in the United States [1]. This number has been steadily increasing over the last two decades, likely due to increased life expectancy and, more significantly, the obesity epidemic. Traditionally, this procedure has been performed under general anesthesia. However, neuraxial and regional anesthesia have become more commonly utilized to aid in postoperative analgesia and reduce the side effects of opioids, namely sedation, nausea, and vomiting. Postoperative pain control has a significant impact on earlier ambulation, initiation of physical therapy, better functional recovery, and overall patient satisfaction [2]. Moreover, optimal pain management can reduce the duration of hospitalization and the risk of adverse events, such as deep vein thrombosis [2]. Regardless of the anesthetic type, multimodal analgesia is often utilized, including NSAIDS, acetaminophen, and opioids.

Complete analgesia for hip arthroplasty is achieved with blockade of the femoral nerve, obturator nerve, nerve to the quadratus femoris, superior gluteal nerve, and sciatic nerve. Individually, anesthetizing these nerves is cumbersome. Single-shot injections that provide adequate coverage include the lumbar plexus (or psoas compartment) block, the femoral nerve block, the fascia iliaca compartment block, and the quadratus lumborum block [3]. Though several of these regional anesthesia techniques are efficacious in providing adequate postoperative pain relief, they do have the potential of causing motor weakness of the quadriceps muscles, thus limiting ambulation. A relatively new block, the erector spinae plane block (ESPB), has been described to be effective in providing analgesia to the hip without motor blockade of the quadriceps muscles.

The ESPB block was first described in the literature in 2016 when it was used to treat chronic neuropathic thoracic pain. Since then, there have been studies demonstrating its efficacy with a thoracic approach for analgesia in breast surgery and rib fractures [4]. Only a few case reports to date have demonstrated a lumbar approach to achieve analgesia for hip arthroplasty [5–7]. Two major benefits to this block are its relatively low risk of complications due to its anatomical position [5], as well as the lack of risk of mechanical nerve damage as there is no direct contact with any nerves [6].

In this study, we tested the hypothesis that the addition of a lumbar ESPB to spinal anesthesia is superior to spinal alone in postoperative pain management in patients undergoing elective primary total hip arthroplasty. The primary objective was to evaluate postoperative opioid consumption at 24 hours between the two groups. Secondary outcomes included a comparison of opioid consumption at 8 hours and 48 hours postoperatively, as well as a comparison of median pain scores at 24 hours and 48 hours post surgery.

## 2. Materials and Methods

### 2.1. Study Design

This was a randomized, prospective, single-blind, single-center clinical trial conducted at Montefiore Medical Center, registered at ClinicalTrials.gov (NCT03801863). The study was approved by the Albert Einstein College of Medicine Institutional Review Board (IRB: 2018-9687). Written informed consent was obtained from all participating patients before inclusion in the study. An independent data and safety monitoring board oversaw the study's conduct and reviewed blinded safety data.

### 2.2. Patient Recruitment

Patients were recruited from March 2019 to July 2021. Inclusion criteria included patients between the ages of 18 and 80 years undergoing elective primary unilateral total hip arthroplasty with spinal anesthesia by participating surgeon co-investigators. The principal investigator initially screened participants for eligibility; phone calls, recruitment, enrollment, and written consent were obtained by the research staff. Exclusion criteria included patient refusal, inability to understand and sign consent, allergy or hypersensitivity to any of the study medications, chronic opioid use (daily opioid use for greater than 1 month), chronic gabapentin/pregabalin use (routine use for greater than 1 month), use of more than 2 antipsychotic medications, contraindication to neuraxial anesthesia, thrombocytopenia (platelets <100,000/mCL), coagulopathy (INR >1.4 or insufficient time since stopping systemic anticoagulation), body mass index (BMI) ≥50 kg/m^2^, anterior surgical approach, and patients with American Society of Anesthesiologists (ASA) IV or V classification.

### 2.3. Randomization and Blinding

Patients were randomized in a 1 : 1 ratio using computer-generated randomization into the control group (no block) or the ESPB group, the latter of which received a preoperative lumbar ESPB with 30 ml of 0.375% ropivacaine. A statistician who was not involved in the analysis of the data prepared the randomization schedule. A research staff member who was not directly involved in the study prepared the randomization envelopes. On the day of the surgery, the regional anesthesiologist was provided a sequentially numbered sealed envelope containing the assignment and then performed the block accordingly, thus being unblinded, in addition to the intraoperative anesthesiologist and the patients, as the envelopes were opened in their presence. The research assistants enrolling and collecting data were blinded.

### 2.4. Protocol

All patients received oral acetaminophen 975 mg and oral pregabalin 50–100 mg preoperatively. Patients in the ESPB group then received lumbar ESPB at either the*L*2, *L*3, or *L*4 transverse process on the ipsilateral surgical side following premedication at the direction of the regional anesthesiologist. The level at which injection was performed was determined by clear identification of the erector spinae muscle above the transverse process.

Intraoperatively, all patients received a spinal anesthetic consisting of 0.5% bupivacaine (2.4 ml–3 ml) + 15 mcg intrathecal fentanyl with moderate intravenous (IV) sedation (a maximum of 4 mg of midazolam and 200 mcg of fentanyl with propofol and/or dexmedetomidine infusion titrated to effect). Intravenous ketorolac 15–30 mg was administered for multimodal analgesia.

Providers ordered IV hydromorphone 0.2–0.4 mg every 15 minutes for 4 doses as needed (PRN) for severe pain in the postanesthesia care unit (PACU). Patients were discharged from PACU after being able to flex and extend the nonsurgical knee, as documented by the recovery room nurses. Upon admission, multimodal analgesia was initiated in all patients with an IV hydromorphone patient-controlled analgesia (PCA) pump (0.2 mg every 10 minutes), oral acetaminophen 975 mg every 8 hours, oral pregabalin 25–50 mg every 12 hours, and IV ketorolac 15–30 mg every 8 hours for 3 doses. At 8:00 am on postoperative day (POD) 1, the hydromorphone PCA was discontinued, and patients were transitioned to oxycodone 2.5–10 mg every 4 hours PRN. After 3 doses of ketorolac, patients were transitioned to celecoxib 200 mg every 12 hours.

### 2.5. Lumbar Erector Spinae Plane Block Procedure

**Figure 1:**
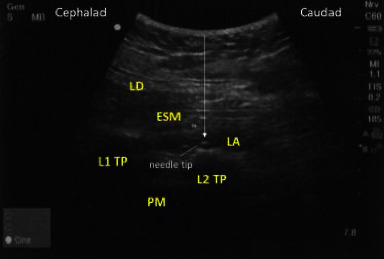
Ultrasound image of lumbar erector spinae plane block. LD = latissimus dorsi; ESM = erector spinae muscle; LA = local anesthetic; *L*1 = lumbar transverse process; *L*2 = lumbar transverse process; PM = psoas muscle. The white arrow indicates needle placement using an out-of-plane technique. The local anesthesia is injected just below the erector spinae muscle and above the transverse process of the targeted vertebral body.

Preoperative lumbar ESPB was performed on the ipsilateral surgical side under conscious sedation (0–2 mg IV midazolam and 0–100 mcg IV fentanyl). The block was performed under a sterile technique with the use of a SonoSite Edge II ultrasound with a curvilinear transducer (5–2 MHz, rC60xi) and a 22-gauge, 3.5-in needle (Chiba). The patient was placed in the lateral decubitus position with the operative side facing up. The transducer was placed in a parasagittal plane along the spinous process. After identifying the spinous process of the *L*4 vertebrae, the probe was moved laterally to identify the *L*4 transverse process. If the *L*4 level was not easily visible, the ultrasound probe was moved cephalad to identify either the *L*3 or *L*2 transverse processes. Using an out-of-plane technique, the needle was inserted and advanced until making contact with the transverse process, after which it was withdrawn slightly as shown in (Figure 1). Once negative aspiration was confirmed, 30 ml of 0.375% ropivacaine was administered. A craniocaudal spread of local anesthetic provided confirmation of appropriate needle position. Block success was assessed by the loss of cold sensation in the posterolateral distribution of the hip 15 minutes after block placement. All block procedures were performed by or under the supervision of an experienced regional anesthesiologist.

### 2.6. Outcomes

The primary outcome measure was cumulative opioid consumption at 24 hours after PACU arrival reported in oral morphine equivalents (OME). Secondary outcomes included total opioid consumption at 8 and 48 hours after surgery. Pain scores were assessed using the numerical rating scale (NRS). All data were collected from the electronic medical record (EMR), as documented by nurses in the PACU and on the admitting floor. Other outcomes assessed were the length of hospital stay, adverse events as related to the block, and the presence of quadriceps weakness documented by the physical therapist on their first assessment (within 4 hours of inpatient admission) and the Acute Pain Service provider.

### 2.7. Statistical Analysis

#### 2.7.1. Sample Size Calculation

The primary objective of this superiority study was to compare the 24 hour opioid consumption between the two groups. Our clinical audit and the published literature reported that 29 mg ± 15 mg of OME were used in the first 24 hours. The study was powered to demonstrate the minimal clinically significant difference of 33% in 24-hour opioid consumption between the control and block groups. Based on a two-sided alpha of 0.05 and a type II error of 20% to achieve a clinically significant 33% difference, a total of 88 subjects were needed for the study.

#### 2.7.2. Data Analysis

The normality of the data distribution was analyzed using the Shapiro-Wilk test. All baseline and clinical patient characteristics were summarized as medians (interquartile range) for continuous variables and the number (%) of patients for categorical variables. Continuous variables were analyzed using the Mann–Whitney–Wilcoxon test, while the chi-square test or Fisher's exact test was used for categorical variables. Reported *p*-values are unadjusted and not corrected for multiple comparisons. For all analyses, two-sided tests were used, and *p* < 0.05 was taken to indicate significance. All analyses were conducted using an intention-to-treat approach. Data analyses were performed using SPSS software (ver. 25.0; SPSS Inc., Chicago, IL).

## 3. Results

**Figure 2:**
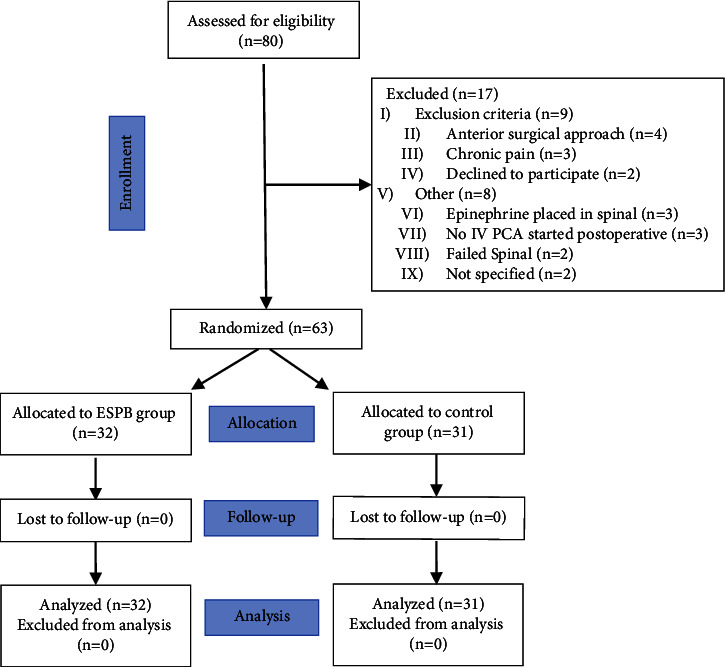
CONSORT diagram of the study.

From February 2019 to July 2021, 80 patients scheduled for elective total hip arthroplasty were assessed for eligibility. Sixty-three patients met eligibility criteria; 31 and 32 patients were randomized to the control group and the ESPB group, respectively (Figure 2). Baseline patient characteristics were similar between the two groups, as described in Table 1. The study was interrupted for 7 months due to the pause in elective surgical procedures during the COVID-19 pandemic in New York, which began in late March 2020. The study resumed in November 2020; however, it was terminated prematurely primarily due to a significant reduction in patient recruitment due to the increased number of same-day joint replacements (<24 hours stay).

**Figure 3:**
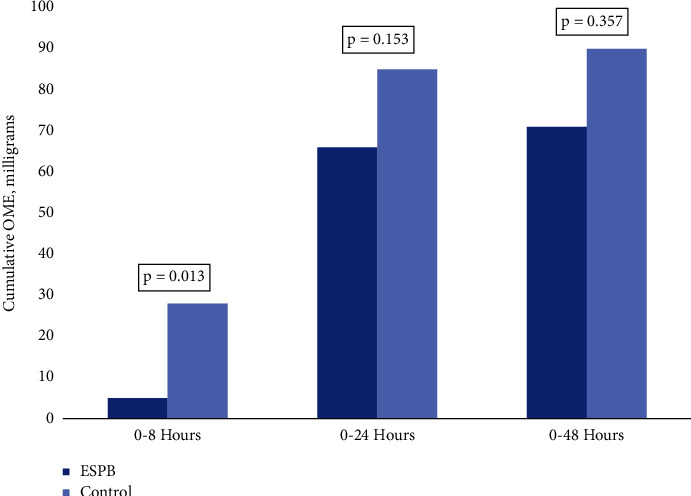
Postoperative opioid consumption.

**Figure 4:**
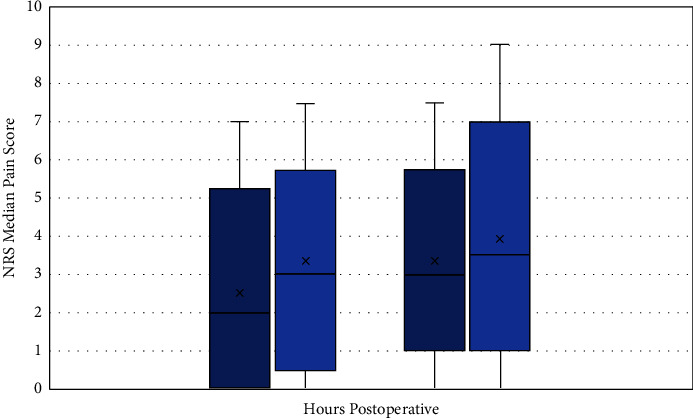
Postoperative pain scores.

The primary objective failed to demonstrate the superiority of the combination of the ESPB with spinal compared to spinal alone on 24-hour postoperative opioid consumption. The median [*Q* 1, *Q* 3] opioid consumption was 66 [38, 105] mg and 85 [61, 135] mg OME in the ESPB group and the control group, respectively (*p* = 0.153) (Figure 3). However, the control group had significantly higher median opioid consumption than the ESPB group in the first 8 hours postoperatively, 28 [8–44] mg OME versus 5 [0–20] mg OME (*p* = 0.013). There was no statistically significant difference in opioid consumption between the two groups at 48 hours postoperative (71 [57, 141] mg and 90 [42, 150] mg OME in the ESPB group and the control group, respectively) (*p* = 0.357). Additionally, there was no significant difference in pain scores between the two groups at either time point (Figure 4). At 24 hours, median pain scores were 2 [0–3.5] and 3 [1–4] in the ESPB and control groups, respectively (*p* = 0.143). At 48 hours, median pain scores were 3 [2–4] in the ESPB group and 3.5 [2–5] in the control group (*p* = 0.617).

There was no difference in adverse events between the two groups including any need for perioperative blood transfusion. There was also no documentation of the presence of quadriceps weakness or falls by the patient, as documented in the physical therapist note.

## 4. Discussion

In this prospective randomized controlled trial, the addition of lumbar ESPB to spinal anesthesia did not reduce opioid consumption within the first 24 hours (*p*=0.153) or after 48 hours (*p*=0.357) postoperatively as compared to spinal anesthesia alone in patients undergoing elective primary total hip arthroplasty. However, there was a statistically significant benefit observed with reduced opioid consumption in the first 8 hours postoperatively (*p*=0.013). There was no statistically significant difference in pain scores between the two groups at 24 or 48 hours. Our study results demonstrate a linear trend in the decrease in opioids used by the ESPB group. In fact, the control group used almost 22% more opioids in the first 24 hours, which supports the fact that a subset of patients likely exists who might benefit from this regional anesthesia technique. Additionally, the wider dispersion of opioid use data suggests an unaccounted-for heterogeneity in opioid use. The absolute difference in opioid use at 8 and 24 hours, however, is worth further exploration.

This is one of the few randomized controlled trials to evaluate the use of lumbar ESPB for analgesia in total hip arthroplasty. In terms of efficacy, our results are consistent with recent case reports and observational studies of ESPB utilized in hip surgeries, only one of which pertains specifically to total hip arthroplasties [5–9]. The most similar study was performed by Ahiskalioglu et al., who demonstrated that deposition of 40 ml of a local anesthetic mixture (20 ml 0.5% bupivacaine, 10 ml lidocaine 2%, and 10 ml normal saline) between the erector spinae and *L*4 transverse process for both hemiarthroplasties and intramedullary femur nailing resulted in adequate analgesia with a median time of 8 hours [8]. This is similar to our findings of decreased opioid requirements in the first 8 hours postoperatively, despite the use of a different local anesthetic.

As previously mentioned, postoperative analgesia for hip arthroplasty is achieved with blockade of the femoral nerve, obturator nerve, nerve to the quadratus femoris, superior gluteal nerve, and sciatic nerve. Lumbar plexus (or psoas compartment) block, femoral nerve block, fascia iliaca compartment block, quadratus lumborum block, and the erector spinae plane block (ESPB) are blocks that can provide adequate analgesia to the hip [3]. While the lumbar plexus block is efficacious, it is technically difficult to perform; additionally, as the needle is advanced into the deep muscles, it has a relatively high potential for systemic toxicity [10]. The femoral nerve block is simple to perform and is commonly used for analgesia for hip fractures. However, a successful femoral nerve block leads to quadriceps muscle weakness [11], which makes it less ideal for patients receiving hip arthroplasty as early ambulation and physical therapy are often encouraged only hours into the postoperative period. The fascia iliaca block is also relatively easy to perform, but as with the femoral nerve block, quadriceps weakness is a known side effect if done using an infrainguinal approach [3]. Depending on the approach, the quadratus lumborum block can successfully provide a sensory block between T6-L3 without causing motor weakness (which can occur with the injection of local anesthetic via the anterior approach) [3, 12–15]. In comparison to the aforementioned blocks, the ESPB seems to be the only block to provide analgesia without documented motor blockade of the quadriceps muscles.

The ESPB is believed to be efficacious due to the substantial spread of local anesthetic. The aforementioned study performed by Ahiskaligolu et al. utilizing magnetic resonance imaging demonstrated the spread of the local anesthetic mixture with the contrast between the *T*12 and *L*5 transverse processes and erector spinae muscle, as well as between the multifidus muscle and iliocostal muscle at the *L*2–*L*4 levels [8]. The contrast was also observed anterior to the transverse process, spreading to the paravertebral, foraminal, and (partially) epidural spaces, as well as the region where the lumbar nerves enter the psoas muscle. Conversely, cadaveric studies of the lumbar erector spinae plane block have reported little to no diffusion of the local anesthetic into the paravertebral space and ventral rami, as well as limited craniocaudal spread [16, 17].

Our study has several limitations. One of the major limitations is in regard to blinding. The patient and the regional anesthesiologist (and, subsequently, the surgeons, nurses, and acute pain provider) became unblinded upon randomization as only the ESPB group received a block. An alternative approach to maintaining blindness could have involved the regional anesthesiologist performing a block on all study patients and administering a sham injection for the control group. In this scenario, an unblinded research assistant that was not involved in the patient assessment or data collection could have provided an unlabeled syringe of 30 cc of local anesthetic or saline to the regional anesthesiologist, depending on which group the patient had been randomized to. This approach was not considered, as we felt that putting the control group at risk for procedural complications as well as the added discomfort of performing a block as a placebo was unethical.

Another limitation of our study was inconsistent documentation of block success. The intention was for sensory testing to be performed 15 minutes after block completion, but there were several instances in which the patient was taken to the operating room immediately after block completion in an attempt to maintain the scheduled surgical start time. It is, thus, unclear how many blocks included in the analysis were actually functional. Additionally, of those blocks that were assessed prior to surgery, we saw an inconsistent sensory loss in the *L*1–*L*3 dermatomal distribution. This was thought to be due to an inconsistent spread of local anesthetic cephalad as a result of the anatomic position of the lower spine muscles, though previous studies demonstrated that the spread of local anesthetic from a lumbar ESPB on contrast imaging extends up to *T*12 [8, 16, 17]. In addition, the ESPB was also done at three different transverse processes between *L*2–*L*4 due to inconsistent layering of the spine muscles.

A third limitation is related to the local anesthetic used for the study. The duration of action of perineural ropivacaine ranges from 5 to 12 hours [18], consistent with our results of statistically less opioid consumption in the ESPB group at 8 hours but not at 24 hours after arrival in the PACU post procedure.

Additional study limitations include the fact that this was a single-center trial (which may attenuate the external validity) and that the surgical procedure was performed by three different surgeons with some variability in technique, although they all utilized the posterior approach to hip replacement. Lastly, the study was interrupted due to the pause in elective surgical procedures during the COVID-19 pandemic, and was terminated prematurely due to a significant reduction in patient recruitment; there was a shift at our institution from inpatient total hip arthroplasty to same-day surgery to avoid hospital admissions. A new standardized enhanced recovery pathway was also created for total hip replacements to facilitate same-day ambulatory surgery. This pathway included the use of quadratus lumborum block type I (lateral quadratus lumborum block) and other nonregional anesthesia multimodal pain strategies for postoperative pain management. Therefore, the goal number of recruited patients was not obtained, which may have contributed to the lack of statistical significance.

## 5. Conclusion

In summary, our study shows a potential benefit of lumbar ESPB in reducing opioid requirements in the first 8 hours after hip arthroplasty but not thereafter. Considering the small sample size and wider dispersion of the opioid data, our results at 24 hours are inconclusive. Therefore, a larger cohort randomized trial is needed to evaluate the opioid requirements after adjusting for potential confounders. Our study results also warrant further exploration of ESPBs with the addition of local anesthetic prolonging adjuncts such as dexamethasone, dexmedetomidine, buprenorphine, or a longer-acting local anesthetic.

## Figures and Tables

**Table 1:** Baseline characteristics of study participants.

	ESPB group (*n* = 32)	Control group (*n* = 31)	*p* value
Age	62 [50, 74]	60 [49, 71]	0.820

Sex			0.859
Male	11 (17.5%)	10 (15.9%)	
Female	21 (33.3%)	21 (33.3%)	

BMI	33.9 [27.9, 40.0]	33.2 [24.1, 42.3]	0.271

Preoperative marijuana use	3 (4.8%)	2 (3.2%)	0.548
Medical history			
Diabetes mellitus	11 (17.5%)	5 (7.9%)	0.096
Osteoporosis	2 (3.2%)	4 (6.3%)	0.368
Sciatica	20 (58.8%)	26 (65.0%)	0.680
Herniated disc	2 (3.2%)	1 (1.6%)	0.573
Case time (minutes)	115 [82, 148]	118 [88, 148]	0.441
Length of stay (days)	2 [1, 3]	3 [1, 5]	0.389

Categorical variables are reported as frequencies (proportions); continuous variables, as median [interquartile range]. ESPB, erector spinae plane block; BMI, body mass index.

## Data Availability

Data are available upon request to the corresponding author. (The institution review board need to be contacted first by Dr. Nair. Please email him. Singh Nair, MD, Ph.D. Research Assistant sinair@montefiore.org).
